# Sahel terrorist crisis and development priorities: case of financial allocations for the control of non-communicable diseases in Burkina Faso

**DOI:** 10.3389/fpubh.2023.1253123

**Published:** 2023-10-12

**Authors:** Moussa Ouedraogo, Dia Sanou, Ines Wendlassida Zaheira Kere, Souleymane Sankara, Nana Thiombiano-Coulibaly, Ousmane Ouedraogo, Bassibila Zoungrana, Fatoumata Hama-Ba, Aly Savadogo

**Affiliations:** ^1^Doctoral School of Sciences and Technology, Laboratory of Applied Biochemistry and Immunology (LABIA), Joseph Ki-ZERBO University, Ouagadougou, Burkina Faso; ^2^Nutrition Directorate, Ministry of Health, Ouagadougou, Burkina Faso; ^3^FAO, Subregional Office, Eastern Africa, Addis Ababa, Ethiopia; ^4^United Nations Children's Fund (UNICEF), Ouagadougou, Burkina Faso; ^5^Food Technology Department, Institute for Research in Applied Sciences and Technologies, National Center for Scientific and Technological Research, Ouagadougou, Burkina Faso

**Keywords:** terrorism, financing, budget allocation, NCDs, Burkina Faso

## Abstract

In Africa, nearly 46% of all mortality will be attributed to non-communicable diseases (NCDs) by 2030. While the cost of inaction far exceeds the cost of action against NCDs, global funding for the prevention and control of NCDs is minimal. The objective of this was to explore the Ministry of Health budget allocations for NCDs from 2010 to 2020 as well as the effect of the terrorism crisis on these allocations. The methodology was based on the budget tracking tool developed by the Scaling Up Nutrition Movement. Twenty-nine budget lines related to the prevention and/or control of NCDs have been identified. About 29.9 million USD were allocated to the fight against NCDs with an absorption rate of more than 98%.There is an upward trend of allocated budget characterized by an exponential increase from the development of the national integrated strategic plan for the fight against NCDs (2016–2020). In 2017, an increase of 184% compared to 2016 was observed. However, the efforts were challenged by the emergence of the terrorist threat which triggered in January 2016, leading to a drastic reduction in allocations for NCDs in favor likely of defense and security priorities as well as addressing the needs of internally displaced persons. A trend analysis suggests that the NCDs budget significantly decrease as the country global terrorist index increase. Further analysis is needed to better understand the implication on NCD incidence, and identify advocacy opportunities for mitigating the negative impact of the terrorist treat on NCDs and other development issues.

## 1. Introduction

Non-communicable diseases (NCDs) are a major cause of poverty and thus a serious threat to the achievement of the Sustainable Development Goals (SDGs) (1). They are biggest silent killer, causing 9 million deaths each year among people under 60 years in low- and middle-income countries. That represents a slow-motion development emergency (2). In sub-Saharan Africa, NCDs are expected to be the leading cause of death by 2030 (3). For all countries, the cost of inaction far exceeds the cost of action against noncommunicable diseases (4). It is encouraging to see that NCDs are now gaining momentum in the global development agenda. In September 2011, world leaders agreed on a roadmap with concrete commitments, to tackle the global burden of NCDs (5). While much efforts has been made globally, significant progress in the fight against NCDs has been observed only in high-income countries (6). This is mainly due to the fact that funding from the global community for the prevention and control of NCDs in developing countries remains insufficient – overshadowed by donor support for communicable diseases, maternal and child health and other traditional health issues (7, 8). The total cost of implementing a combination of individual and population-wide interventions, in terms of health expenditure, amounts to 4% of GDP in low-income countries, and less than 1% in upper-income countries (4). Ouedraogo et al. reported that many concerns around the governance of NCDs, including issues of funding remain unanswered in most ECOWAS countries (9). There is a consensus that adequate financing is a powerful catalyst for Scaling Up the fight against NCDs. Overall, there has been a downward trend in the Official development assistance (ODA) to address NCDs over the past decade (10). In addition, there is a significant mismatch between international resources and the real needs of recipient countries. Some authors argue that the NCD target (Target 3.4) of the Sustainable Development Goals (SDGs) cannot be achieved if funding does not increase (11). With the high likelihood that the burden of NCDs will likely increase in coming years, countries should prioritize funding for their prevention and control.

In 2015, Burkina Faso adopted an Integrated Strategic Plan for the Fight against Non-Communicable Diseases 2016–2020 with a total implementation cost of $9 million. However, this plan has not been evaluated to appraise the effectiveness of the implementation of the planned interventions.

Furthermore, the challenging security situation that the country is experiencing since 2016 could have a considerable impact on the budget allocations for NCDs (12). While this could negatively affect investment priorities, little is known about the implications for social sectors such as prevention and control of NCDs. Most of the available data focus on the number of incidents and the immediate effects on lives lost, the economy and different socio-economic indicators such as health and nutrition, access to water and health services, education. The objective of this study is to examine the budget allocations of the Ministry of Health of Burkina Faso for the fight against NCDs between 2010 and 2020 and to assess the effects of the security crisis related to terrorist threat on these allocations.

## 2. Methodology

The methodological approach is based on the methods developed by the Scaling Up Nutrition Movement (13) to track budget allocations and expenditures for nutrition, adapted by UNICEF and Action Against Hunger (14). This approach has been developed for tracking nutrition investments by identifying budget lines and subsequent allocations related to nutrition specific and nutrition-sensitive interventions. The method comprises 4 fundamental steps: planning, data collection, validation, and data analysis. Although the tool makes it possible to monitor both funding from technical and financial partners (external funding) and the government budget (internal funding), only state allocations were analyzed in this study due to the difficulty in obtaining exact data on funding from technical and financial partners. The budget lines considered in this analysis are those related to the prevention and control of non-communicable diseases (NCDs) as indicated by the Ministry of Health. While NCDs should be addressed by multiple sectors to facilitate the sharing of the financial burden to promote joint accountability for achieving specific NCD-related targets, the exploratory study focus on allocations from the health sector. The lack of a common multi-sectoral results framework listing consensual interventions for the fight against NCDs in Burkina Faso makes financial monitoring difficult in the other contributing sectors.

### 2.1. Data source

The analysis focused on the allocations and final expenditure of the Ministry of Health of Burkina Faso during the period 2010 to 2020. This data was retrieved from the Expenditures Integrated Circuit (CID – circuit intégré de la dépense) platform of the Ministry of Economy, Finance and Development (MINEFID). The platform includes all operations containing allocations and expenditures as well as transfers made by the government to local authorities and other public institutions. At the request of the research team, an annual database (2010 to 2020) was extracted from the CID by a MINEFID agent, containing the allocations and expenditures and then made available to authors for the various analyses. The authors then carried out a meticulous examination of the budget of the Ministry of Health to extract the budget lines relating to NCDs.

Information on the terrorism index was obtained from the various annual reports on the Global Terrorism Index of the Institute for Economics and Peace (15).

### 2.2. Data collection and processing (extraction of budget lines)

Data from the CID platform were scrutinized to extract all budget lines relating to NCDs. Certain budget lines were not taken into account in accordance with the SUN approach used. Indeed, the methodology suggests that budget lines should not be included in the analysis when they are related to:

- the payment of the salaries of public officials;
- the operation of the general and technical departments; hospitals, health districts, and training institutions, etc.;
- the organizations of exams and competitions;
- operating and project expenses.

### 2.3. Validation of budget lines

**Figure 1:**
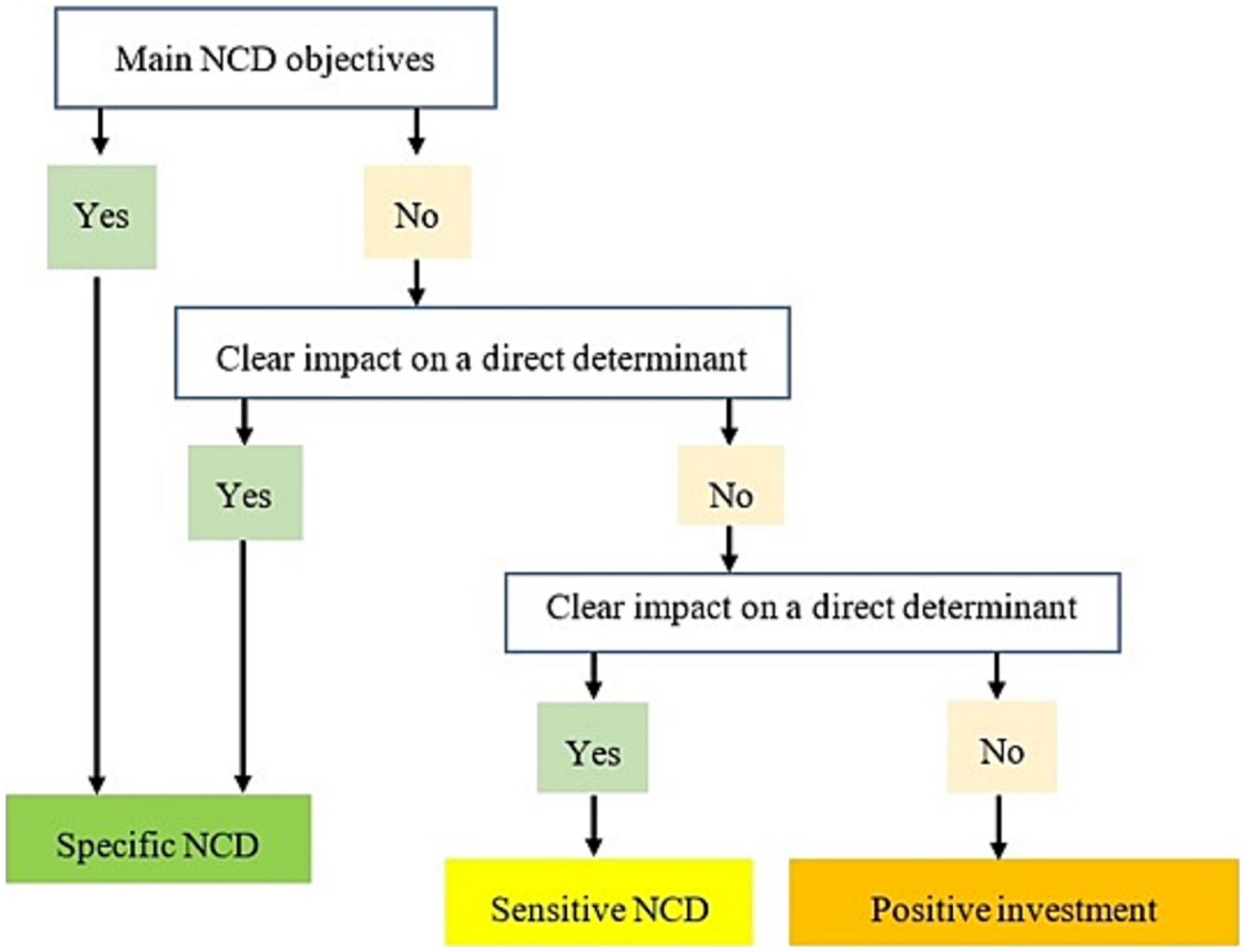
Conceptual framework for categorizing pro-NCD budget lines adapted from the conceptual framework for categorizing pro-nutrition budget lines (14).

The authors analyzed the budget lines selected during the data collection phase in order to carry out the categorization and weighting. The categorization made it possible to classify the budget lines selected into 3 categories namely specific, sensitive, and positive (Figure 1). Then, the validated budget lines were weighted. This involved assigning a rate to each line according to its estimated level of contribution to the prevention and fight against NCDs in the country. This rate was determined by the authors based on available scientific evidence, the current status of the intervention implemented in the country, the context of NCDs at the national and international level, and finally on the basis of the interventions proposed in the Action Plan prevention and control of non-communicable diseases 2013–2020 (4, 16). Budget lines considered specific to NCD were given a weighting of 100%. As for those classified as sensitive to NCDs, three levels of weighting were applied according to the estimated degree of sensitivity of the investments, i.e., 10, 25 and 50% for investments with low, medium and high sensitivity, respectively. Favorable investments were not taken into account in total NCDs expenditures. As a result, a zero rate was applied to these lines.

### 2.4. Data processing and analysis

The database obtained was processed with the Stata 12 software. Data were summarized using descriptive analyses and frequency calculation for budget allocations. Trends of budgetary allocations was explored throughout the years and according to the overall index of terrorism in Burkina Faso from 2015 to 2020. The Pearson correlation coefficient was used to assess the correlation between the proportions of allocations and the overall terrorism index. The selected budget lines were categorized according to the objectives of the WHO global action plan for the fight against NCDs 2013–2020. The differences observed were evaluated using the student test (*T*-Test) with a significance level of 5% and a confidence interval of 95%.

## 3. Results

### 3.1. Budget lines

Overall, twenty-nine (29) budget lines related to the prevention and/or management of NCDs in Burkina Faso were identified. Following the categorization and weighting, three (3) budget lines were considered “specific” to NCDs, eighteen (18) were “sensitive” and eight (8) were considered to be “positive investments” (Table 1).

**Table 1:** Selected budget lines.

*N*°	Budget lines	Allocation type	Categorization
1	Childbirth & Emergency Obstetric Care / Subsidies to other beneficiary categories	Current transfer	Positive investment
2	Acquire contraceptive products	Investment	Positive investment
3	Acquire vaccines and consumables	Investment	Sensitive
4	Acquire micronutrients	Investment	Positive investment
5	Acquisition of Hospital Equipment / STATE / CHR / Technical equipment-tools	Investment	Positive investment
6	Support Dialysis Units/Subsidies to other categories of beneficiaries	Investment	Specific
7	Ensure the construction and equipment of the Infrastructures of the Bobo-Dioulasso hemodialysis project	Investment	Sensitive
8	Ensure coverage of community-based health workers/Subsidies to other categories of beneficiaries	Current transfer	Sensitive
9	Ensure Burkina’s commitments to Global Fund financing for the management of certain diseases/Subsidies to other categories of beneficiaries	Current transfer	Sensitive
10	Ensure the medical examination of workers	Current transfer	Sensitive
11	National Center for Apparatus and Orthopedics/ Subsidies to other beneficiary categories	Current transfer	Positive investment
12	National Center for the Fight against Blindness/ Subsidies to other beneficiary categories	Current transfer	Sensible
13	Build and equip a cancer center in Ouagadougou/Research and development costs	Investment	Sensible
14	Female cancer screening	Current transfer	Specific
15	Availability of maternal health services / STATE / Eta tranche / Other purchases of goods & services	Investment	Sensible
16	Students in 6th year of Pharmacy / Current transfers to households	Current transfer	Sensible
17	End of medical cycle students	Current transfer	Sensible
18	Free preventive care/Subsidies to other beneficiary categories	Current transfer	Positive investment
19	Hospital interns/Routine transfers to households	Current transfer	Sensible
20	National Vaccination Days/ Subsidy to public establishments	Current transfer	Sensible
21	Tobacco control/Transfers to supranational organizations & government contribution	Current transfer	Specific
22	Doctors in specialization/Routine transfers to households	Current transfer	Sensible
23	Standardization of health facilities / STATE / Standardization	Investment	Positive investment
24	Workers’ Health Office/subsidies to other beneficiary categories	Current transfer	Sensible
25	Pay Burkina’s contribution to the World Health Organization	Current transfer	Sensible
26	Pay Burkina’s contribution to the West African Health Organization/Transfers to supranational authorities and contributions to international organizations	Current transfer	Sensible
27	Support the construction of the Tengandogo radiotherapy center	Investment	Sensible
28	Social programs/STATE/Dialysis unit/Other purchases of goods & services	Investment	Sensible
29	Care for children aged 0 to 5/Subsidies to other beneficiary categories	Current transfer	Positive investment

### 3.2. Budget allocations and expenditures

Over the 11 years period considered (2010 to 2020) i.e., the Ministry of Health of Burkina Faso has allocated nearly 17.33 billion FCFA (29.9 million US dollars) representing an average of 2.72 million dollars per year in the fight against NCDs. This allocation represents about 1.55% of the total budget of the Ministry of Health during the same period. The budget absorption rate, which was defined as the percentage of allocated budget effectively used was more than 98% (Table 2). With this high absorption rate, we carried out the analyses with only budget allocations.

**Table 2:** Budget allocations and expenditure related to NCDs of the Ministry in charge of Health from 2010–2020.

Year (2010–2020)	Total	Yearly average
Total budget allocations for NCDs (FCFA)	29885033.32^a^ $	2716821 $
Total expenses for NCDs (FCFA)	29459619.47 $	2678147.22 $
Absorption rate (expenses / allocations in %)	98.58%	98.58%

a 1 dollar US = 580 FCFA.

### 3.3. Allocation by intervention type

**Figure 2:**
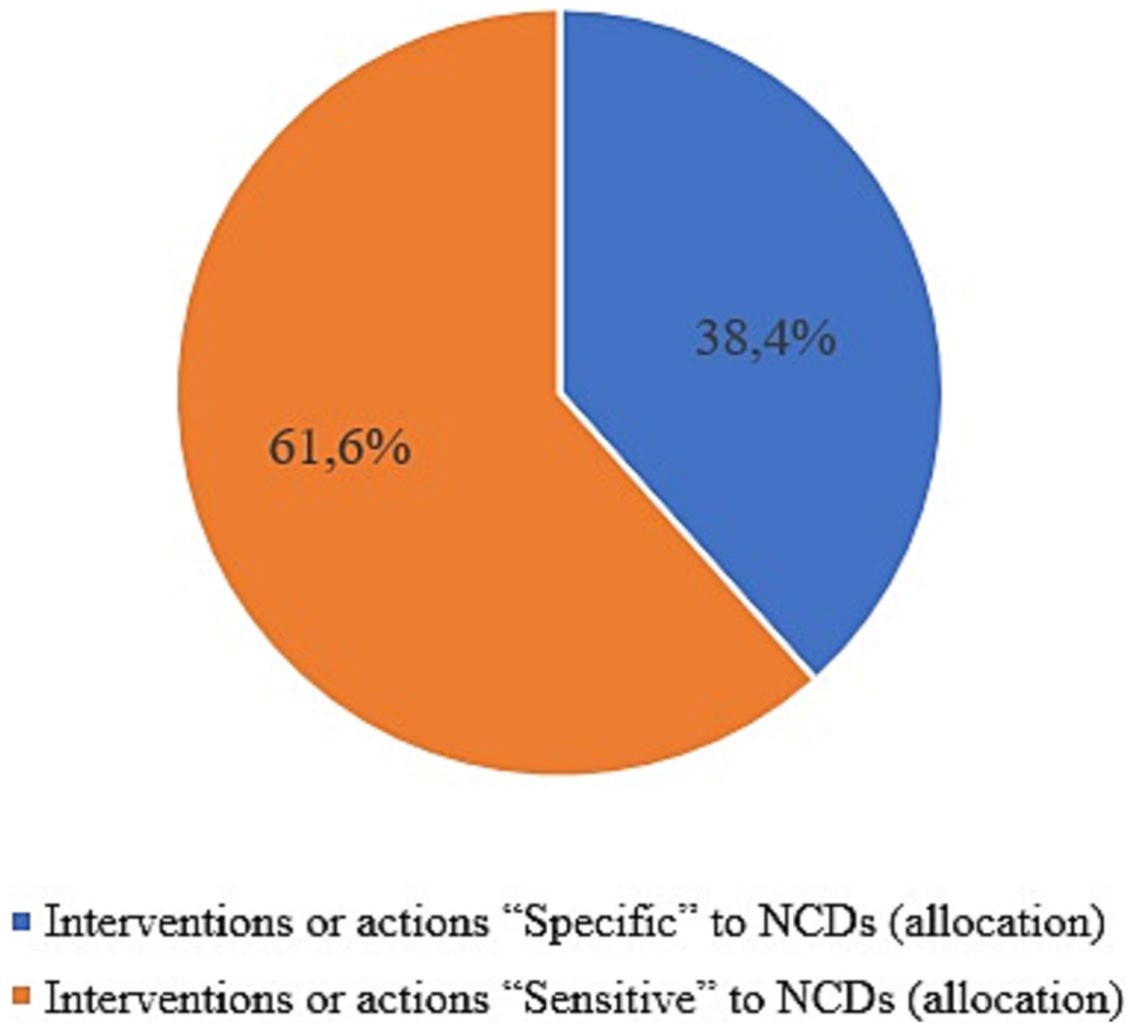
Allocation by intervention type.

Of this budget of 17.33 billion de FCFA (29.8 million $), 6.64 billion representing 38.4% were allocated to “NCDs specific” interventions, while 10.68 billion, i.e., 61.6% were allocated to “sensitive” interventions (Figure 2).

### 3.4. Budget allocation by objectives of the global action plan

**Figure 3:**
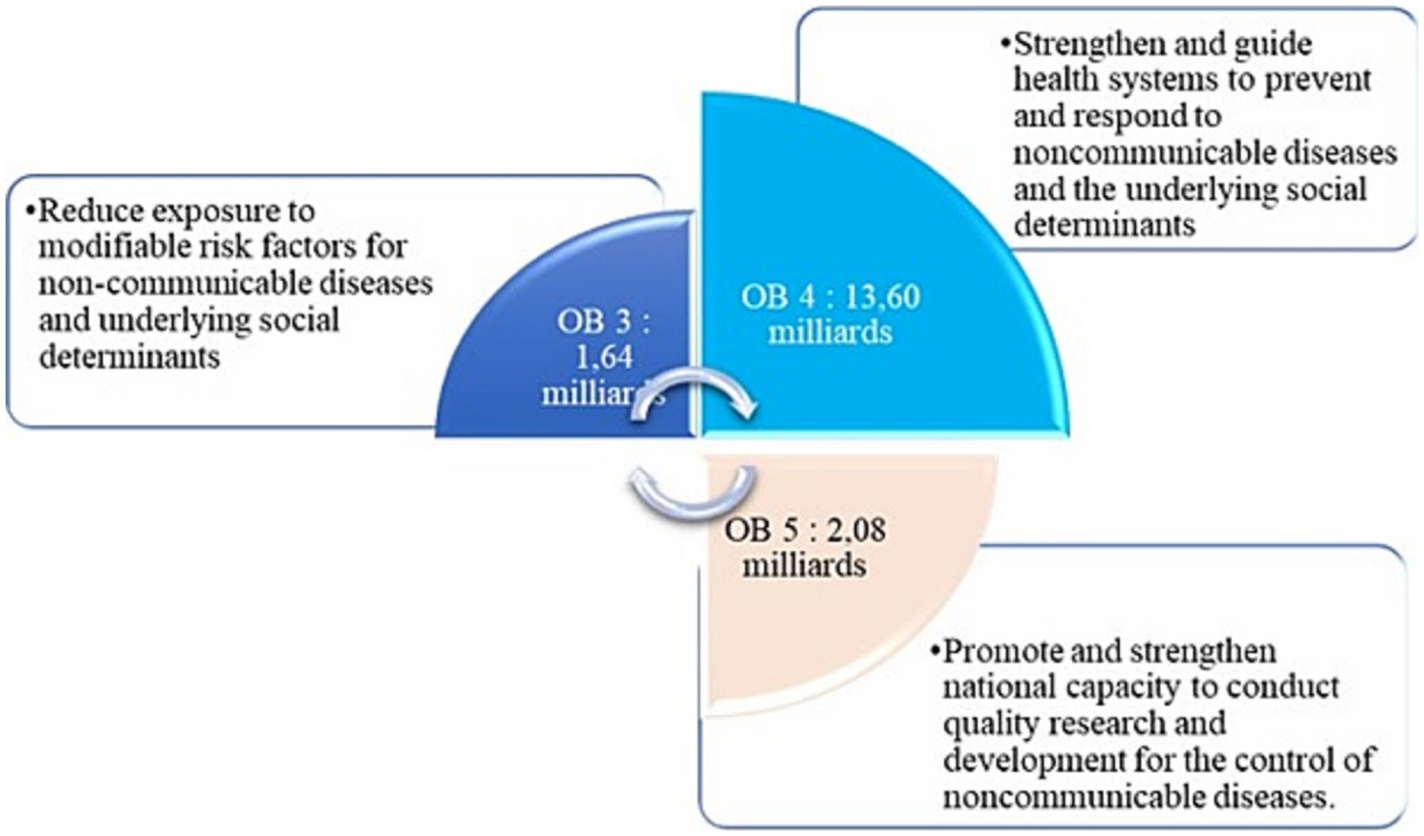
Allocations by objectives of the global NCD action plan.

The analysis of the budget according to the objectives of the WHO global action plan for the fight against NCDs 2013–2020 suggests that only interventions aimed at achieving 3 out of the 6 WHO objectives, namely objectives 3, 4 and 5 received budget allocation (Figure 3). Objective 4, which aims to strengthen and guide health systems, received most of the allocation, i.e., 13.6 billion, i.e., 78.48% of total funding. Objective 5 which aims to promote and strengthen the national capacity to carry out actions to prevent and fight against NCDs was the second most funded with 2.08 billion, approximately 12% and finally Objective 3 which aims to reduce the exposure to modifiable risk factors received 1.64 billion, i.e., 9.5%.

The action plan Objectives which did not receive any budgetary allocation include Objective 1 “Give higher priority to the fight against noncommunicable diseases in the global, regional and national agendas and in the development goals agreed at the at the international level, by strengthening international cooperation and awareness,” Objective 2 “Strengthen national capacities, leadership, governance, multisectoral action, and partnerships to accelerate the fight against noncommunicable diseases in the countries “and the Objective 6 “Monitor trends and determinants of noncommunicable diseases and assess progress in prevention and control.”

### 3.5. Evolution of budget allocations from 2010 to 2020

**Figure 4:**
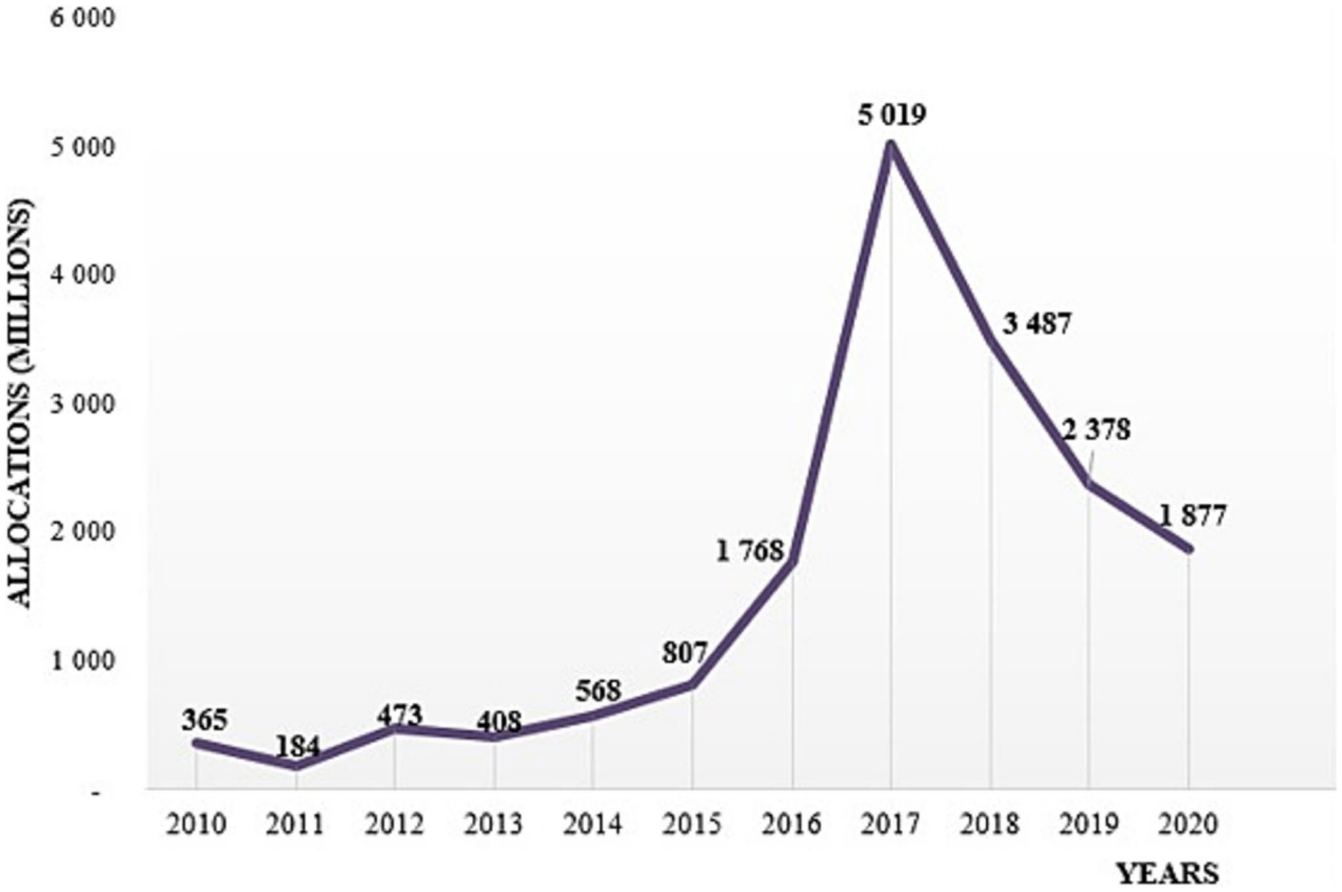
Evolution of budget allocations from 2010 to 2020.

The trend analysis of allocations shows an upward trend in annual allocations from 365 million in 2010 to more than 5 billion in 2017, with a serrated evolution between 2010 and 2013 (Figure 4).

The allocations then experienced a drastic reduction of more than 62%, down from 5.019 billion in 2017 to 1.877 billion in 2020, i.e., almost its 2016 value.

### 3.6. Allocations before and after the development of the integrated strategic plan for the fight against NCDs 2016–2020

In 2015, the Ministry of Health developed an integrated national strategic plan to fight against NCDs 2016–2020. Immediately after the adoption of this Integrated Strategic Plan, the annual budget for NCDs more than doubled from 807 million CFA francs in 2015 to 1.87 billion CFA francs in 2020 (Figure 4). There is a funding peak to 5.019 billion in 2017, which represents an increase of 522% compared to 2015, the year preceding the entry into force of the National Strategic Plan 2016–2020.

**Figure 5:**
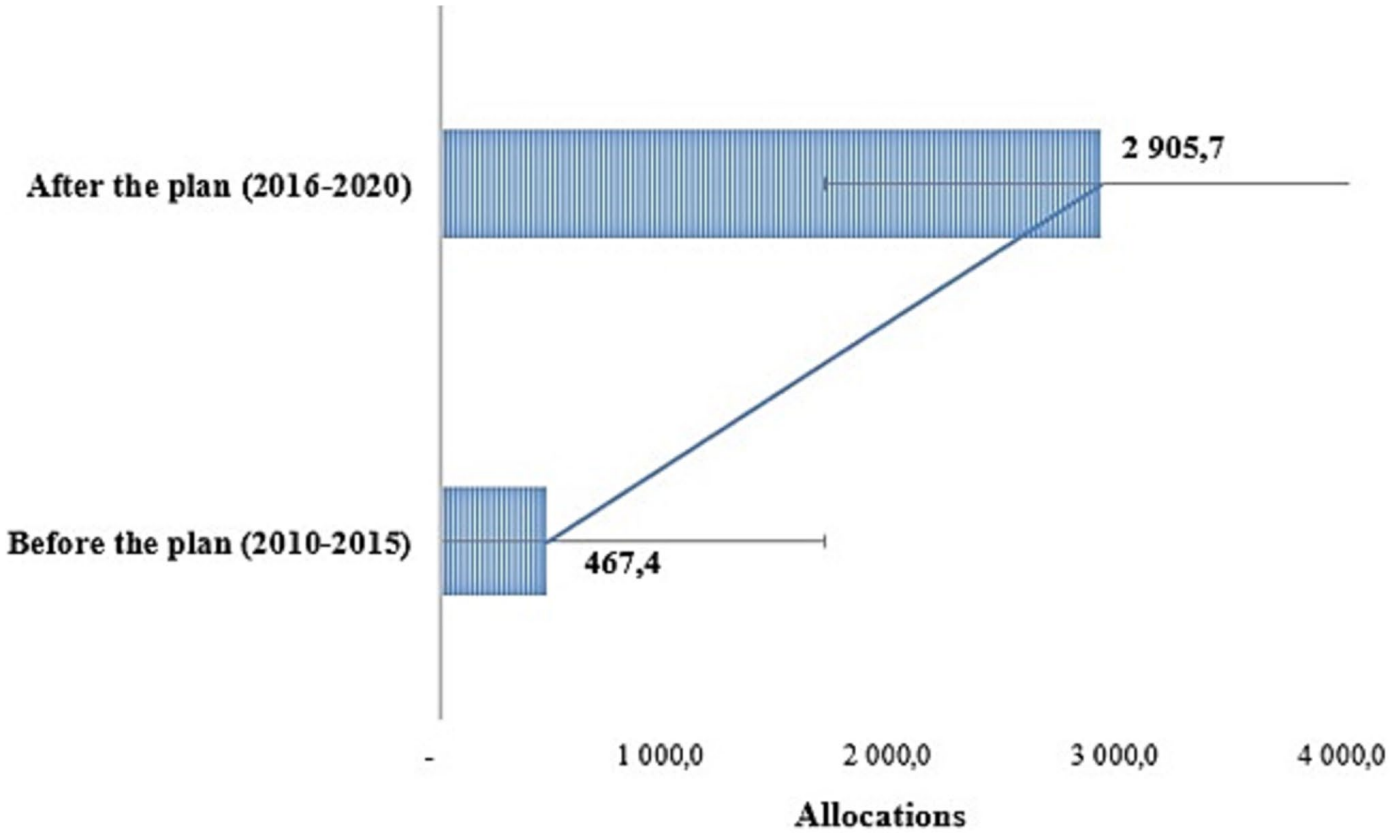
Comparison of average allocations before and after the strategic plan.

The comparison of the average annual budget allocations before (2010–2015) and after (2016–2020) the development of the strategic plan (Figure 5) shows that overall, the budget allocations improved after the development of the strategic plan. Indeed, the average annual allocation increased significantly (*p* = 0.02) from about 467.4 million before the adoption of the Strategic Plan to more than 2.9 billion FCFA afterwards.

### 3.7. Effect of terrorism on budget allocations

On January 15, 2016, a double terrorist attack was perpetrated by al-Qaeda in the Islamic Maghreb, killing in total 30 person including 20 expatriates. Since that year the security situation has continued to deteriorate with government losing control of over 40% of the territory resulting in thousands of people internally displaced and many health facilities closed or operating at a minimum.

**Figure 6:**
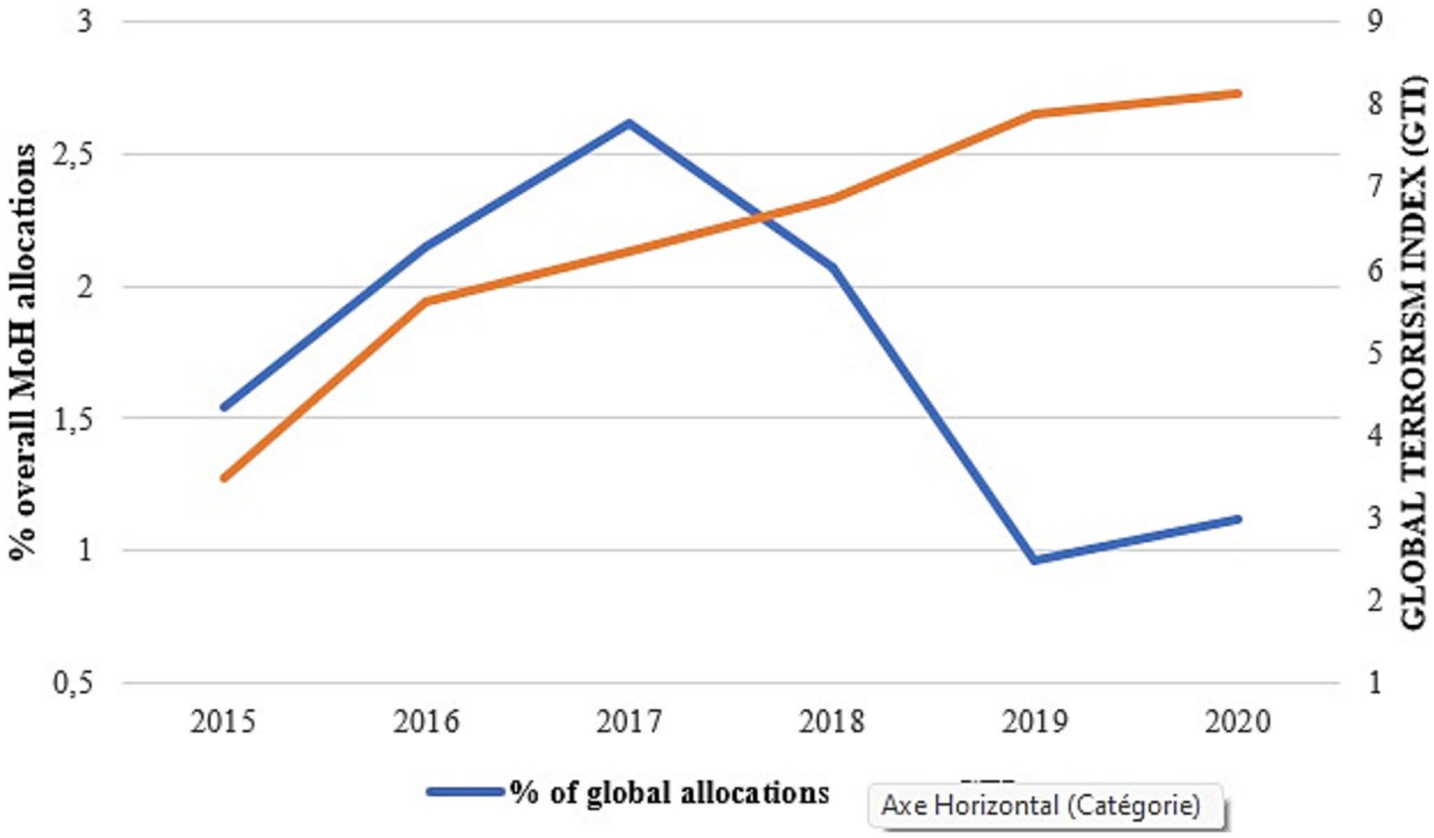
Comparative trends between MoH allocation for NCDs and global terrorist index.

Figure 5 shows that the proportion of the budget directed to NCDs increased until 2017, when terrorist attacks began to intensify with an overall terrorism index of 6.2. From 2017, there was a gradual reduction in Ministry of Health allocations for NCDs as the number of terrorist incidents increased and became widespread across the country (Figure 6). Thus, we observe a significant negative correlation between the global terrorism index and the Ministry of Health allocations for NCDs (*r*^2^ = −0.32, *p* = 0.008).

## 4. Discussion

This study contributed to documenting one of the most important evidence gaps related to governance of NCDs in low-income countries, i.e., level of investments for the prevention and control of NCDs. Findings show that over the period 2010–2020, the Burkina Faso Ministry of Health allocated nearly 1.5% of its sectoral budget for the control of NCDs. The financial allocation increased by more than 119% in 2016 compared to 2015, the year before the adoption of the integrated national plan (2016–2020) with a record increase of more than 522% in 2017 compared to 2015. Unfortunately, the emergence and expansion of the terrorism threat in the region reversed the government efforts, and the financial allocation for NCD keep decreasing as the terrorism index increased.

Five years after the endorsement of the Global action plan for the prevention and control of noncommunicable diseases 2013–2030 which committed Heads of State to commit to establishing and strengthening multisectoral national policies and plans for the prevention and control of NCDs, the government of Burkina Faso developed an integrated strategic plan for the fight against NCDs 2016–2020 (5). This is an indication that Burkina leaders acknowledged the devastating impact of noncommunicable diseases in the national agenda and committed to implement WHO recommendations to reduce the NCDs burden. It is well recognized that strong political leadership and commitment at the highest national level is the first key action to furthering a development issue on government agendas and galvanize actions. A policy framework is a tool to translate commitment into action through a set of feasible actions and interventions for which specific and timed targets and indicators can be developed, and progress measured. While the development of a reference document is not enough to address NCDs, the important increase in budgetary allocations for NCDs suggest that policy adoption can accelerate the scale-up of proven effective actions for the prevention and control of NCDs (17). Although we are unable to clearly establish the impact of the strategic plan on the increase in allocations, the process of developing the national strategic framework has help better understood the problem, well-structured and cost the response, and therefore clarify investment opportunities (17–19). The plan highlight the importance of “screening for female cancer” which is considered one of the most cost-effective interventions in the fight against NCDs with a cost-effectiveness ratio ≤ 1 $ according to WHO (16). The cost of this intervention alone was estimated to 1.5 billion, and thus significantly increasing the national allocation.

The budget allocation was not equitably distributed across the 6 objectives of the WHO action plan, with interventions aimed at achieving objectives 1, 2, and 6. The objective non prioritized are mostly related to enabling environment including agenda setting and governance (Objective 1), capacity strengthening (Objective 2) and monitoring, evaluation and learning (Objective 6). However, while health systems strengthening and the reduction of modifiable risk factors for noncommunicable diseases and underlying social determinants are critical, the impact on the nine NCD targets will be stronger if all six objectives of the global plan are funded (16). The lack of internal resources for evaluations, in particular the carrying out of STEPS surveys, does not make it possible to monitor progress in the prevention and fight against NCDs on the one hand and on the other hand to inform decision-makers with conclusive data. and updated in a timely manner. For example, over the analysis period (2010 to 2020), Burkina Faso carried out a STEPS survey in 2013 thanks to the resources of technical and financial partners compared to 11 nutritional surveys with the SMART methodology.

Findings suggest that after continuous increase over the past year, the annual financial allocation of the Ministry of Health for NCDs reverted in 2017, one year after Burkina Faso experienced its first terrorist attack. Further, after 2017, the allocations for NCDs decreased continuously as the number of terrorist attacks became widespread across the country. There was a strong negative correlation between the global terrorist index and the annual financial allocation for NCDs. It is to be noted that the impact of terrorism on budget allocation was observed in the 2018 budget likely because it was considered minor treat in the first year and the government spending in the response took effect only in the financial planning of 2018. Since then, the security situation continued to deteriorate, and terrorist related insecurity expanded across the country with many implications.

First the government has to increase the financial allocations for defense and security priorities. This has likely resulted in a shift towards reduction in the budgetary allocations of certain ministerial departments and development priorities.

Second not only does the insecurity inflict significant human and material losses, but it can generate innumerable humanitarian disasters (massive displacement of populations, food insecurity and malnutrition, ill health, economic, and social insecurity). Indeed, the increasing insecurity in some part of Burkina Faso led to population displacement towards less risky areas, causing enormous pressure on social infrastructure and financial demand to address the immediate basic social needs (clothing, housing, water, food, and health). The permanent secretariat of the National Council for Emergency Relief and Rehabilitation (SP/CONASUR) estimated that more than 2 million people in Burkina Faso were internally displaced (IDPs) in march 2023 (20). According to the results of CH, close to 2.2 million people need immediate assistance (population in phase 3 to 5) in March 2023 in Burkina Faso (21).

Thirdly, terrorist related insecurity can also yield negative consequences on the economies, including loss of national income, slower economic growth, lower foreign direct investment (FDI) and disparate effects on international trade. The terrorist attacks are said to have reduced net foreign direct investment in Spain by 13.5% and in Greece by 11.9% from the mid-1970s to 1991 (12). These economic losses could negatively affect investment priorities in the social sectors such as health. Terrorist incidents can also reduce investor confidence which leads to a reduction in foreign direct investment in the national economy (22, 23). The Institute for Economics and Peace (IEP), for example, estimated that between 2007 and 2016, foreign direct investment in target countries fell by 43%, from US$4.84 billion in 2007 to $2.74 billion in 2016 and the contribution of foreign direct investment to GDP fell from an average of 3.2% in 2007 to 2.5% in 2016 (23).

The impact of these terrorist attacks on the burden of NCDs is not well documented, yet there is likelihood that NCDs situation may worsen. Druetz et al. (24) have reported that the terrorist crisis lead to a deterioration of indicators of access to maternal health services in Burkina Faso. In Nigeria, other authors observed negative effects of Boko Haram attacks on the likelihood of any antenatal care visit, delivery at a health facility, and delivery attended by a skilled health professional (25). In the current study, we could not obtain reliable quality data on the prevalence of NCDs in Burkina Faso that are regularly collected to assess the impact of both the financial resource allocations and absorption and the negative impact of terrorism attacks on the occurrence of NCDs. The most reliable information on NCD in Burkina Faso was the STEP survey conducted in 2010. The second STEPs was completed in 2022 after we completed our data collection. We considered DHIS2, a web-based platform used as a health management information system worldwide; unfortunately, these data contain a lot of gaps to be used in this type of analysis. As a result, we decided to focus the current analyses on budget allocations, while exploring other opportunities to deepen the analysis to include NCD outcomes.

Beyond this study, lack of or poor data quality is generally one key gap for accountability in health programming as for many development issues where there is consistent budget allocation and absorption without measurement of outcome. Planning and budget allocation for development should therefore account not only for financial resource for the collection of outcome data, but also using the outcome data information for feedback and learning to identify key bottlenecks and gaps and make necessary corrective measures. There is a need to build a comprehensive health information management system, which includes outcome data to foster accountability.

In addition to the challenge of fighting insecurity, many efforts are still needed to improve budget allocations for the fight against NCDs when we know the financial burden of NCDs on households in low-and middle-income countries like Burkina Faso. If the downward trends continue, it may worsen the growing burden of non-communicable diseases, and therefore put a great strain on health systems and the country will not be able to meet the WHO/SDG targets for NCDs.

## 5. Conclusion

This study shows that the government of Burkina Faso through its Ministry of health allocates less than 2% of the health budget for the prevention and control of NCDs even if these diseases are considered a priority health problem and a leading cause of death globally. However, a significant increase in budget allocations has been observed since the endorsement of the WHO Global NCD Action Plan 2013–2020. The domestication of this plan in the national policy landscape through the adoption of an Integrated Strategic Plan for the fight against NCDs (2016–2020) has strengthened the political commitment as evidence by the important increased in the financial allocations for NCDs. Unfortunately, the efforts were reversed by the emergence of terrorist related insecurity triggered by the attacks of 2016 with an exponential reduction in allocations from 2017. To our knowledge, this is the first study in ECOWAS region to that attempts to assess the financial allocation for SDGs. It represents important baseline information to track funding allocations for the control of NCDs over years.

However, the study has some the limitations that may affect the interpretation of findings and are therefore worth highlighting. In this study, due to methodological constraints such as lack of a consensual list of interventions for the fight against NCDs and data availability in other sectors, we limit the budget appraisal to the Ministry of Health. Further study that will include budget allocations from other sectors are needed. In addition, the classification and weighting methodology used to estimate the contribution rate for the different budget lines is derived from the one used for nutrition.

Finally, while there is evidence of negative impact of the terrorist related insecurity on financial allocations for NCDs, it is not clear how this will translate in terms of NCD indicator. Further analysis is needed to better understand the implication on NCD incidence and identify advocacy opportunities for mitigating the negative impact of the terrorist threat on NCDs and other development issues, which might be killing equally or even more than the terrorist crisis.

## Data availability statement

The original contributions presented in the study are included in the article/Supplementary material, further inquiries can be directed to the corresponding author.

## Author contributions

MO, DS, AS, and NT-C collaborated for the conception and design of the study. MO led data collection in collaboration with IK and SS. MO and IK organized the database. MO carried out the statistical analysis. MO, DS, and OO interpreted the data. MO wrote the first draft of the manuscript under the guidance of DS and AS. All authors contributed to the article and approved the submitted version.
